# A short review of the most common safety concerns regarding creatine ingestion

**DOI:** 10.3389/fnut.2025.1682746

**Published:** 2025-12-01

**Authors:** Igor Longobardi, Marina Yazigi Solis, Hamilton Roschel, Bruno Gualano

**Affiliations:** 1 Applied Physiology and Nutrition Research Group, School of Physical Education and Sport, Faculdade de Medicina FMUSP, Universidade de São Paulo, São Paulo, Brazil; 2 Center of Lifestyle Medicine, Faculdade de Medicina FMUSP, Universidade de São Paulo, São Paulo, Brazil; 3 Laboratory of Assessment and Conditioning in Rheumatology, Hospital das Clínicas HCFMUSP, Faculdade de Medicina FMUSP, Universidade de São Paulo, São Paulo, Brazil; 4 Division of Rheumatology, Hospital das Clinicas HCFMUSP, Faculdade de Medicina, Universidade de São Paulo, São Paulo, Brazil

**Keywords:** dietary supplements, nutrition, sports, creatinine, adverse effects, pregnancy, malignancy

## Abstract

Creatine monohydrate supplementation is widely used for enhancing athletic performance and improving clinical outcomes, but concerns regarding its safety persist, particularly relating to risk of cancer, impaired kidney function, dehydration, and gastrointestinal issues. This short review critically evaluates these concerns based on current scientific evidence. Although some theoretical risks, such as creatine’s potential to form carcinogenic compounds, have been discussed, the available research does not support a link between creatine supplementation and cancer. In terms of kidney health, studies consistently show no adverse effects on renal function in healthy individuals, though caution is advised for those with pre-existing kidney conditions and pregnant women, as evidence is lacking for these populations. Claims that creatine leads to dehydration or muscle cramps during exercise are largely unsupported by controlled studies, which demonstrate no significant effects on hydration or thermoregulation; in fact, creatine may reduce the incidence of muscle cramps and assist in maintaining thermoregulatory balance. Gastrointestinal distress is reported in some individuals, particularly at high doses, but such effects are dose-dependent and not universally experienced. Overall, the evidence suggests that creatine monohydrate supplementation is generally safe when used appropriately, with further research needed to understand its impact on specific populations.

## 1 Introduction

Since its rise in popularity as a performance-enhancing supplement, creatine has been subject to intense scrutiny regarding its safety. Reports of potential adverse effects, including associations with cancer, kidney dysfunction, dehydration, and gastrointestinal issues, have fueled debates among researchers, clinicians, and consumers. However, many of these concerns stem from early anecdotal reports or misinterpretations of physiological mechanisms. This short narrative review critically examines the evidence surrounding these concerns, assessing their validity in light of the latest scientific findings. By addressing both theoretical risks and empirical data, this paper aims to provide a balanced perspective on the safety profile of creatine monohydrate supplementation, particularly in special populations, including pregnant women.

## 2 Risk of cancer

It has been suggested that creatine may contribute to the formation of chemical compounds, such as heterocyclic amines (HCAs) (1), which are associated with an increased risk of cancer (2). Furthermore, due to its role in cellular metabolism, there is also a current concern that creatine could support cancer progression and metastasis (3). In contrast, creatine and cyclocreatine (its analog) have been shown to delay tumor growth *in vitro* and animal models and acts as an anticancer agent, working synergistically with chemotherapeutic drugs (4–6). This section will explore the current understanding of both perspectives in this field.

Creatine is a naturally occurring guanidine compound that is endogenously synthesized and/or obtained through the diet, primarily from animal sources (e.g., red meat, fish, and poultry) (7). From an evolutionary perspective, it is noteworthy that creatine has always been a significant component of the human diet, with estimates of prehistoric intake reaching five to ten times the typical levels consumed nowadays (7, 8). It seems then unlikely to imagine that human beings have lost their ability to properly process this nutrient over time. The carcinogenicity of HCAs, which form when creatine combines with glucose and amino acids during high-temperature cooking methods (e.g., grilling and smoking), has been experimentally demonstrated in animal and *in vitro* studies using much higher amounts of HCAs than those found in cooked/processed meats (9). Evidence from human studies corroborating its carcinogenicity remains scarce.

Pereira et al. (10) have investigated the effects of low- and high-dose creatine monohydrate supplementation (ranging from 2 to 20 g/day) on the production of HCAs in healthy humans using a non-counterbalanced single-blind crossover design. Out of 576 assessments performed on 149 urine samples, only nine exhibited quantifiable levels of HCAs (10). Importantly, six of these samples came from placebo-supplemented participants, whilst only the remaining three were from creatine-supplemented participants, suggesting that creatine supplementation did not stimulate the formation of such mutagens in healthy humans. This conclusion aligns with data from the National Health and Nutrition Examination Survey (NHANES), which indicates that higher dietary creatine intake is associated with a lower risk of cancer or malignancy (11, 12).

Conversely, emerging experimental evidence has linked creatine to cancer progression and metastasis, particularly through the phosphocreatine energy shuttle mediated by creatine kinase in cancer cells (3). Human liver metastases from colorectal and pancreatic cancers have been shown to express higher levels of creatine kinase and SLC6A8, the creatine transporter (13). Using orthotopic mouse models, Zhang et al. observed that creatine-treated mice presented with colorectal and breast cancer metastases through Smad2/3 activation (14), a key mediator of the TGF-β signaling pathway, which regulates cell invasion, immune responses, and microenvironment modification (15). These findings seem to support a potential link between creatine metabolism and cancer progression. On the other hand, creatine supplementation has been demonstrated to enhance the activity of CD8 T cells, which play a central role in mediating and orchestrating immune responses against cancer (16). Moreover, a considerable number of studies have shown that creatine and cyclocreatine can inhibit tumor growth (4–6). This effect is believed to result from the restoration of the creatine-phosphocreatine system in cancer cells, as well as the regulation of acidosis, inflammation, and oxidative stress (17). Some researchers even advocate for creatine’s therapeutic potential in cancer, especially when combined with exercise as an adjunct therapy to ameliorate treatment-related side effects and prevent skeletal muscle loss (18). However, the complexity of the diversity of cancer types, tumors, and experimental models, along with the inherent differences between species, limits our ability to predict creatine’ effects in oncological treatments. Further studies are warranted to better understand these interactions.

Therefore, the claim that creatine intake increases the risk of cancer in humans is not substantiated (Table 1). However, many dietary supplements available on the market may contain unapproved pharmaceutical ingredients, including carcinogenic contaminants (19), so the consumption of creatine supplements with unverified purity should be avoided. Additionally, alternative formulations to creatine monohydrate warrant further investigation.

**TABLE 1:** Summary of the evidence on the most common safety concerns regarding creatine monohydrate supplementation.

Safety concern	Conclusions	Risk	Evidence grade*
Cancer	● Safe based on current evidence ● Experimental studies suggest potential anticancer effects ● No human studies support an increased carcinogenicity	Not supported	II
Kidney function	● Safe for healthy individuals ● Individuals with pre-existing kidney conditions should be monitored using non-creatinine biomarkers	Not supported	I
Dehydration/thermoregulation	● Does not impair hydration or thermoregulation ● May help maintain body temperature	Not supported	I
Muscle cramps	● May reduce incidence of muscle cramps during exercise	Not supported	II
Gastrointestinal distress	● Mild and infrequent dose-dependent symptoms ● Splitting doses (≤ 5 g per serving) reduces the risk ● Large trials show no significant difference versus placebo	Unlikely	II
Pregnancy problems	● Limited human evidence ● Animal studies demonstrate consistent protective effects	Unknown	III–IV

Grade I – Good/Strong: consistent evidence with minimal bias; Grade II – Fair: some inconsistencies or limitations, but generally reliable evidence; Grade III – Limited/weak: inconclusive evidence from few or weak studies; Grade IV – Expert Opinion: evidence based on clinical experience, opinion, or extrapolation from basic research; Grade V – Not Assignable: no supporting evidence available. *Academy of Nutrition and Dietetics Evidence. (2022). Grade Definitions and Chart. URL: https://www.andeal.org/content.cfm?content_id=11 Accessed [20 Oct 2025].

## 3 Renal dysfunction

Although creatine is one of the most widely used dietary supplements worldwide, benefiting both physically active (e.g., athletes) and clinical populations, some concerns still persist among users, health professionals, and the general public. This section provides a brief overview of the state-of-the-art regarding the effects of creatine supplementation on kidney health [for a comprehensive review, see reference (20)].

Warnings about the potential harm of creatine supplementation on kidney health first emerged in the late 1990’s, driven by case studies and preclinical trials in various animal models (21–30). However, most of these reports rely on retrospective observational data from individuals with preexisting kidney conditions, who engaged in high-intensity and/or high-volume exercise [which can lead to rhabdomyolysis (31)], and/or who abused other substances, including those known to affect renal function (32, 33). Additionally, as previously discussed, findings from animal studies often cannot be extrapolated to humans, as even closely related species may respond differently to creatine intake (34). Therefore, the methodological limitations of these studies compromise their ecological validity.

The concerns about impairments in kidney function are based on the assumptions that creatine intake directly interferes with creatinine (Crn) metabolism. In fact, creatine supplementation increases the body’s total creatine pool, particularly in skeletal muscle, where it is spontaneously (non-enzymatically) and irreversibly degraded into Crn at a rate of approximately 2% per day (1). Consequently, individuals taking creatine supplements may naturally have higher serum Crn levels, without having any impairment in renal function. The problem is that gold-standard methods for assessing renal function are typically restricted to specialized facilities; thus, serum Crn is often used as a proxy for renal function or to estimate glomerular filtration rate (GFR) as a more accessible alternative (35).

In the 1990’s and the mid-2000’s, Jacques Poortmans and colleagues published a series of original studies employing various experimental designs, ranging from single-group pre-to-post investigations to non-randomized, placebo-controlled crossover trials (36–39). Their findings consistently showed no evidence of renal function impairments based on several parameters (including estimated GFR, serum and urinary Crn, urea, proteinuria, and albuminuria) following a wide range of supplementation protocols (1–80 g/day of creatine monohydrate for durations spanning 5 days to 60 months) (36–39). However, it is important to note that participants in all studies were predominantly healthy young men, which limits the generalizability of the findings. In 2011, a narrative review published by the same research group concluded that “*the few renal incidents* (associated with creatine supplementation) *that have been reported remain largely anecdotal*” (40). Nonetheless, the authors also warned that creatine supplementation may pose a potential risk for individuals at risk for impaired kidney health (e.g., patients with diabetes, hypertension, reduced GFR, etc.) (40). In contrast, a recent pilot study by Bernales-Delmon et al. examined the effects of 8 weeks of creatine supplementation on physical function and body composition in adults undergoing chronic hemodialysis (41). Except for a statistically significant increase in serum creatinine, no changes were observed in laboratory analytes after supplementation (41). Similarly, Chang et al. reported that administering creatine monohydrate before each dialysis session for 4 weeks caused no adverse effects, except for a slight increase in serum creatinine (42).

We also have published several studies investigating the effects of creatine monohydrate supplementation (from 5 to 20 g/day for up to 24 months) on renal function in various populations, including healthy and diseased individuals at risk for impaired kidney health (10, 43–53). We have found no evidence of renal impairment, even in clinical populations, such as a young man with a single kidney and mild renal insufficiency (48), older adults with type 2 diabetes (49), children and middle-aged adults with diverse rheumatic conditions (50–52), and pre-frail and frail older adults (53). These observations have been based on multiple renal function biomarkers that are independent of Crn metabolism (e.g., cystatin C, serum and urinary electrolytes, proteinuria, and albuminuria), including the ^51^Cr-EDTA clearance, which is considered a gold-standard technique for measuring GFR.

Therefore, available literature consistently shows that creatine monohydrate is safe when taken at recommended doses, even in clinical populations (Table 1). However, for individuals with (or at risk of) decreased renal function, close monitoring during creatine supplementation is recommended given the limited data. To prevent misdiagnosis (false positives), renal function should be assessed in creatine users preferably using a combination of biomarkers independent of Crn metabolism, such as direct GFR measurements, cystatin C, proteinuria, albuminuria, and/or urinary albumin-to-creatinine ratio.

## 4 Disturbances in thermoregulation, dehydration and muscle cramps

Another concern about creatine is its putative detrimental effects on thermoregulation and hydration status. Dehydration during exercise triggers a series of physiological changes that can lead to adverse outcomes, including reduced plasma volume, increased cardiac stress (i.e., increased heart rate and reduced stroke volume, accompanied by a reduction in cardiac output), elevated plasma osmolality, increased body temperature, and decreased heat tolerance. These alterations can ultimately compromise athletic performance (54). For years, creatine has been implicated as an allegedly contributing factor leading to the deaths of several football players (55) and wrestlers (56) due to exertional heat stroke.

The physiological rationale underpinning the hypothesis that creatine supplementation could contribute to dehydration and heat stress arises from its osmotic properties. As an osmotically active substance, creatine facilitates water influx into the intracellular space via sodium-dependent creatine transporter. Because this process involves sodium, water is also taken up into the muscle cells, where it helps maintain intracellular osmolality (1), particularly during the initial phase of supplementation. Studies have demonstrated that consuming 20 g/day of creatine for 5 days can result in a 1–3 kg increase in body weight, mostly attributed to total body water (TBW) accumulation (57, 58). Given this osmotic effect, some have hypothesized that creatine may alter fluid balance by promoting intracellular water (ICW) retention, thereby reducing extracellular water (ECW) availability. This shift could, in theory, impair thermoregulation and contribute to an increased incidence of muscle cramps by interfering with the muscle’s contraction-relaxation mechanisms, or by causing dehydration, electrolyte imbalance, and impaired thermoregulation (59). These concerns were overstated after a roundtable report (expert consensus, 2000) from the American College of Sports Medicine (ACSM), which advised caution regarding creatine supplementation in athletes engaged in weight cycling and/or performing strenuous exercise in hot environments, recommending its avoidance under such conditions (60).

However, this proposition is based on very weak evidence, primarily anecdotal reports (from individuals using multiple sports supplements) and speculation that creatine may alter fluid balance or impair thermoregulation. Studies based on self-reports have examined the effects of creatine supplementation among athletes (61–63), particularly football players training in hot environments. Juhn et al. (61) examined 52 male athletes and found that 30% reported diarrhea, 25% muscle cramps, 14% dehydration, and 17% undesired weight gain. Similarly, Greenwood et al. (62) surveyed 219 collegiate athletes, finding that 41% reported creatine use, with 34 athletes experiencing negative effects such as gastrointestinal distress (24%) and muscle cramping (27%). The perceived effects in this study may or may not have been influenced by creatine supplementation, but factors such as training, supplement interactions, nutritional habits, hydration, and heat/humidity could also have affected athletes’ perceptions (62).

Apart from this anecdotal evidence, several controlled studies have examined the effect of creatine supplementation and exercise in hot and/or humid conditions, showing no detrimental effects on thermoregulation and hydration status (64, 65), both in men and woman (66). One of the first studies to examine the effects of creatine under conditions likely to promote dehydration was conducted by Vogel et al. (67). After a 75-min intermittent exercise protocol designed to induce fluid loss, they showed no significant differences between creatine and placebo group in body mass loss or percent change in plasma volume. Regarding hydration measures, multiple studies, but not all (68), have reported increases in TBW and ICW following creatine supplementation, with no difference in sweat rates (69, 70), electrolytes levels (e.g., Na^+^ and K^+^) (71), plasma volume (69, 71), or hematocrit (59). In fact, it has been suggested that these increases in TBW and ICW may contribute to maintaining or enhancing thermoregulation. For example, the study by Greenwood et al. (72) suggested that 4 months of creatine supplementation (0.3 g/kg/day for 5 days, followed by 0.03 g/kg/day for 115 days) may reduce the risk of dehydration, cramping, or muscle injury in football players training and competing in hot and humid conditions. Additionally, other physiological measures, such as body temperature and heart rate, appear to remain unchanged with creatine supplementation (67, 71–73). Some authors (59, 74, 75) have also shown that creatine attenuates the rise in body temperature during exercise in the heat.

Therefore, clinical evidence does not support the link between creatine supplementation and the increased risk of heat illness or impaired thermoregulation, even in the presence of pre-existing dehydration. In a double-blind, placebo-controlled, crossover study, Watson et al. (76) investigated the effects of creatine supplementation on heat tolerance in 12 dehydrated men (∼2.0% body mass loss). After undergoing a dehydration protocol, participants exercised for 80 min. The creatine group exhibited better maintenance of plasma volume during the first 20 min of dehydration; however, no significant differences were shown between groups for dehydration, plasma Na^+^ and K^+^ concentrations. Aerobic performance, body temperature, and physiological strain index also remained similar between groups.

In summary, although anecdotal reports suggest that creatine may cause side effects in athletes exercising in the heat, a body of evidence from controlled studies shows no negative impact on thermoregulatory or hydration status, including body temperature regulation, dehydration percentage, urinary hydration markers, plasma volume, sweat losses or, muscle cramps (64). On the contrary, by promoting increases in total and intracellular body water, creatine supplementation may help support thermoregulation (76, 77) and potentially lower the incidence of cramping (72), particularly in athletes exposed to hot and humid environments (Table 1).

## 5 Gastrointestinal distress

While creatine is generally well-tolerated, some reports suggest that supplementation may cause gastrointestinal (GI) distress in certain individuals (78, 79). Epidemiological studies have identified GI issues as the most commonly reported adverse effects, which include diarrhea, stomach discomfort, bloating, and other symptoms. A likely explanation for these adverse effects is creatine’s osmotic effect: when high single doses (> 10 g) are ingested, part of the compound may remain unabsorbed in the intestine, drawing water into the lumen and accelerating transit. As discussed, an anecdotal study of 52 male athletes found that 30% experienced diarrhea (61), while 24% of 219 collegiate athletes reported GI distress (62). It is important to note that, in both studies, athletes exceeded the recommended maintenance dose (i.e., 2–5 g/day), with some of them taking three to four times the recommended dose, reaching 17–20 g of creatine per day. However, scientific evidence supporting these associations remains limited.

Potential GI side effects were assessed in 59 soccer players randomly assigned to one of three groups: two daily doses of 5 g creatine (Cr5), a single 10 g/day dose (Cr10), or placebo (PL) (80). After 28 days, diarrhea (39%), stomach upset (23.8%), and belching (16.9%) were reported across all groups, including placebo. No significant differences in GI distress were observed between Cr5 and PL. Similarly, no group differences were found for abdominal pain, heartburn, nausea, bloating, vomiting, or constipation. However, Cr10 showed a higher incidence of diarrhea than Cr5 (55.6% vs. 28.6%, *p* < 0.05) and PL (55.6% vs. 35%, *p* < 0.05). The authors attributed this to higher creatine intake in a single dose, which may leave undissolved creatine in the GI tract, increasing water retention via osmotic activity and leading to diarrhea or loose stools (79). These effects may be minimized by limiting single doses to ≤ 5 g or, if higher daily intake is needed, dividing it into smaller doses (e.g., two daily 5 g doses instead of one 10 g dose).

In line with these findings, a systematic review with meta-analysis on creatine supplementation in female participants found no statistically significant effects of supplementation on GI events when dose regimens were stratified as “maintenance dose only,” “combined dose only,” or “loading dose only” (81). Among the 18 studies analyzed, four reported GI symptoms as the reason for intervention dropout, with similar rates observed in both the creatine and placebo groups (five vs. six participants, respectively) (45, 82, 83). Only one study reported increased adverse GI events when symptoms were combined with muscle cramping (*p* < 0.05). Yet, no significant difference was observed between groups in the overall number of adverse events reported (83). Similar findings, in both younger (40, 77) and older (84) male or mixed-sex populations were observed, with GI disturbances primarily associated with higher creatine doses exceeding commonly studied dosing protocols, or concurrent supplementation (62, 80, 85). Supporting this, Kreider et al. evaluated 685 randomized controlled trials with over 26,000 participants and found no significant differences in the prevalence or frequency of GI issues between creatine and placebo groups (5.5% vs. 4.2%, *p* = 0.820) (86). While a slightly higher number of trials reported GI side effects in creatine users, the overall incidence among participants was comparable to placebo, indicating that these effects are uncommon and typically not clinically relevant (86).

It is also speculated that other additives, ingredients, or contaminants generated during industrial production (e.g., sodium sarcosine, cynamide, dicyandiamide, dihydrotriazines creatinine) in some creatine supplements may cause GI discomfort in sensitive individuals (80). This would be a safety issue related to the purity of the supplement, rather than the creatine itself. Moreover, it has been hypothesized that the combination of creatine and caffeine would increase symptoms of GI distress, which might indirectly reduce performance (87, 88). For some individuals, caffeine may increase peristalsis, leading to stomach discomfort and more frequent bowel movements, although caffeine sensitivity is highly individual. In this regard, Vandenberghe et al. (88) reported mild GI distress after 6 days of creatine (0.5 g/kg/day) and creatine (0.5 g/kg/day) plus caffeine (5 mg/kg/day) supplementation in three subjects (∼33%): two during the first 2 days of creatine ingestion and one over the 3 days of creatine plus caffeine supplementation.

While creatine is generally well-tolerated, gastrointestinal upsetting can occur, particularly with high single doses (greater than 10 g) or when combined with other supplements like caffeine. However, evidence linking creatine to GI distress remains limited and inconsistent, with dose regimens and individual variability being key factors (Table 1). To minimize adverse effects, it is recommended to follow dosage protocols and, if needed, split the daily intake into smaller doses (e.g., ≤ 5 g per dose).

## 6 Creatine supplementation during pregnancy

Pregnancy is a period marked by profound physiological and metabolic adaptations aimed at supporting fetal development and preparing the mother for delivery. Among the nutrients involved in energy metabolism, creatine plays a central role as a high-energy phosphate buffer, maintaining cellular ATP homeostasis. The creatine kinase system is particularly relevant in tissues with high and fluctuating energy demands, such as the uterus, placenta, and developing fetal brain (89, 90). Given these characteristics, creatine supplementation has been investigated as a potential strategy to improve maternal and neonatal outcomes. However, its use during pregnancy raises important safety considerations, as evidence in humans remains limited and inconclusive.

Animal studies provide the bulk of knowledge regarding creatine use in gestation. In rodent and other mammalian models, maternal creatine supplementation has consistently been associated with improved neonatal resilience to hypoxic stress, enhanced survival, and preservation of brain, muscle, and other organ functions (90–94). Importantly, no adverse maternal or fetal effects have been documented in these studies, even when supplementation was initiated early in pregnancy and maintained until term. These findings are biologically plausible, as creatine availability during episodes of oxygen deprivation can sustain mitochondrial ATP production, delay energy failure, and reduce tissue injury. This experimental evidence has led to the hypothesis that creatine supplementation could serve as a prophylactic therapy in pregnancies at risk of intrapartum hypoxia, a major cause of perinatal morbidity and mortality (90). Nevertheless, while preclinical data are promising, interspecies differences in placental transport, creatine metabolism, and developmental trajectories preclude direct extrapolation to humans.

Human evidence remains scarce. A frequently cited case report described the course of a pregnant woman affected by a creatine synthesis disorder (i.e., Arginine:Glycine Amidino-Transferase deficiency) who received creatine supplementation throughout gestation. The pregnancy resulted in the birth of a healthy infant without apparent complications, suggesting safety in that particular context (95). However, the anecdotal nature of this observation, combined with its unique clinical background, prevents generalization to healthy pregnant women. Beyond isolated reports, the current knowledge is derived primarily from observational studies investigating dietary creatine intake and maternal metabolite profiles.

One line of evidence suggests that suboptimal dietary creatine intake may be associated with higher risks of adverse obstetric outcomes. For instance, analyses of maternal dietary patterns indicate that low intake of creatine-rich foods, such as meat and fish, correlates with greater incidence of complications, including low birth weight (96). Observational data indicate that dietary creatine intake below recommended levels may be associated with higher risks of certain obstetric conditions, but the study did not address supplementation safety (96). In addition, there is evidence that maternal plasma creatine concentrations correlate positively with protein intake, reflecting the importance of exogenous dietary sources (97). These associations highlight the relevance of creatine homeostasis during pregnancy but cannot be interpreted as evidence for the safety of supplementation, as observational data are subject to nutritional assessment bias, residual confounding and reverse causation.

Data indicate that plasma creatine concentrations remain relatively stable throughout pregnancy, while urinary excretion declines toward term; guanidinoacetate concentrations, in contrast, fluctuate significantly, suggesting adaptations in endogenous creatine biosynthesis (97). These findings illustrate the complexity of maternal creatine regulation, which appears to adjust throughout pregnancy

Despite the biological rationale and encouraging animal data (90, 91, 93), the absence of randomized controlled trials in humans remains the most significant barrier to recommending creatine supplementation during pregnancy. Regulatory authorities have not established safety categories for creatine in this context, and expert reviews consistently conclude that the evidence base is insufficient. Although creatine monohydrate is considered safe in healthy adults at conventional doses, pregnancy involves unique physiological alterations, including expanded plasma volume, increased renal clearance, hormonal changes, and altered nutrient transfer through the placenta, that could modify both the pharmacokinetics and safety profile of supplementation. Without carefully designed clinical trials, it is impossible to determine whether maternal or fetal risks might emerge from exogenous creatine administration.

Acceptability and attitude studies provide further insight into the potential translation of this intervention. Many pregnant women would be receptive to creatine supplementation if recommended by their healthcare providers, particularly if framed as a strategy to reduce risks of perinatal hypoxia. At the same time, both patients and professionals emphasize the need for robust safety and efficacy data before clinical adoption (98, 99). This aligns with the broader principle that interventions in pregnancy must adhere to the highest evidentiary standards, given the ethical responsibility to safeguard both maternal and fetal health.

In summary, creatine supplementation during pregnancy remains an investigational concept with promising preclinical support but limited human evidence (Table 1). Animal studies demonstrate consistent protective effects against perinatal hypoxia without adverse outcomes (90). However, these findings cannot substitute for randomized controlled trials, which are essential to evaluate safety, efficacy, and appropriate dosing in pregnant women. Until such studies are conducted, creatine supplementation should not be recommended in clinical practice outside of research protocols. The priority moving forward is to design carefully controlled trials that can clarify whether creatine may serve as a safe and effective adjunct in maternal-fetal medicine, particularly for pregnancies at risk of hypoxic complications (90).

## 7 Conclusion

This narrative review on over 30 years of accumulated evidence concludes that:

1. When used at recommended doses (5–20 g/day), creatine monohydrate is safe for most populations and has well-documented performance and clinical benefits.
2. Current evidence does not support a link between its supplementation and increased cancer risk nor renal dysfunction in humans.
3. Consumers should be cautious about supplement purity: potential risks stem from contaminants rather than creatine; therefore, only high-quality, third-party tested supplements should be used, and alternative formulations avoided.
4. Individuals with pre-existing kidney conditions should be monitored closely, as evidence is incipient and limited in this population.
5. Assessment of kidney function should use biomarkers independent of Crn (e.g., direct GFR measurements, cystatin C, proteinuria, albuminuria, and/or urinary albumin-to-creatinine ratio).
6. Available evidence does not support claims that creatine increases dehydration, heat illness, or muscle cramps; actually, it may support thermoregulation and reduce the incidence of cramps in some exercise settings.
7. GI distress is generally mild and infrequent, often occurring with high single doses (> 10 g per dose) or co-ingestion with other supplements (e.g., caffeine).
8. Splitting doses into smaller amounts (e.g., ≤ 5 g per dose) can minimize potential GI discomfort.
9. Animal studies demonstrate consistent protective effects against pregnancy problems; however, evidence in humans is considerably limited.
10. Future research should focus on vulnerable populations (e.g., individuals with or at risk of pre-existing kidney disease and pregnant women).
